# COVID-19 Isolation and Risk of Death in Cyprus Elderly People

**DOI:** 10.3389/fmed.2021.717692

**Published:** 2021-08-02

**Authors:** Marios Kyriazis, George Mikellides, Heraklis Pantelidakis, Marina Polycarpou, Barnabas Panayiotou

**Affiliations:** ^1^National Gerontology Centre, Larnaca, Cyprus; ^2^Materia Group, Nicosia, Cyprus; ^3^Postgraduate School of Medicine, Keele University, Stoke-on-Trent, Staffordshire, United Kingdom

**Keywords:** COVID-19, social isolation, older people, death risk, Cyprus elderly, prevention of mortality

## Abstract

Social isolation is associated with a higher risk of morbidity and death in older people. The quarantine and social distancing measures due to Covid-19 imposed in most countries and particularly in Cyprus, aim to isolate individuals from direct contact with others. This has resulted in vulnerable older people being isolated at their places of residence for several months, while the recommendations for continuing lockdowns do not appear to be ending. The risk of death from causes other than those related to Covid-19 increases in such individuals and it is due to the effects of social isolation. We estimate that in the next years, there will be a significant increase in the death numbers of such older people in Cyprus. The health authorities must develop a program of support for these older individuals to include medical, social, physical, and psychological elements. Examples of such support are given here.

## Introduction

As the spread of Severe Acute Respiratory Syndrome Coronavirus-2 (SARS-CoV-2) continues to affect Cyprus, the elderly population has remained, and it is likely to remain, in enforced isolation for a long time. This is in line with similar policies in other countries. For example, the recommendation in the United Kingdom has been that people aged 70 years and older should be isolated at home for several months (1). However, it is well-accepted that social isolation in such age groups is a significant risk factor for morbidity and mortality and is therefore a serious public health concern (2, 3). This isolation exacerbates a variety of problems affecting older people which includes cardiovascular, cognitive, autoimmune, psychological, and hormonal disturbances (4).

We already knew before the current pandemic that there is consistent evidence linking social isolation and loneliness to worse cardiovascular and mental health outcomes (5). Moreover, we knew in advance that living alone and social disengagement is associated with a 24–32% higher hazard for hospital admission for elderly people suffering from respiratory disease (6).

In a recent qualitative study in Cyprus (7), it was confirmed that social isolation during the Covid-19 era is directly correlated with loneliness, a negative emotion associated with a perceived gap between existing relationships and desired ones. Strongest risk factors for morbidity and death were loss of a loved one, inactive lifestyle even before Covid-19, and clinical depression.

We are already beginning to experience increases in the numbers of non-Covid-19 deaths, compared to previous years (8). Estimates indicate a 17.7% increase in the number of deaths in the USA during 2020, compared to those in 2019, with heart disease and cancer-related deaths being the top two causes (9). It is possible that social isolation, associated with missed hospital treatments and other reasons connected to the strict lockdown measures, contributed to such an increase in deaths (10).

## Morbidity and Mortality

Valtorta et al. (11), examined 5,397 people aged 50 years and above, for an average period of 5.4 years. They found that there was an association between loneliness and increased risk of cardiovascular disease. Their conclusion was that: “*Loneliness is associated with an increased risk of developing coronary heart disease and stroke, independently of traditional cardiovascular disease risk factors. Our findings suggest that primary prevention strategies targeting loneliness could help to prevent cardiovascular disease.”* Another study of 479,054 people over 7 years (12) confirmed the above findings concluding that “*Isolated and lonely persons are at increased risk of acute myocardial infarction (AMI) and stroke, and, among those with a history of AMI or stroke, increased risk of death.”*

In a study of 6,500 participants aged 52 years and above, selected from The English Longitudinal Study of Aging in the UK (13), it was reported that social isolation (a state of complete or near-complete lack of contact between an individual and society) was associated with elevated risk of mortality (Hazzard Ratio of 1.26). Other studies also found significant association between loneliness (a temporary and involuntary lack of contact with others) and increased mortality (14). The increased mortality is not only linked to cardiovascular conditions. For example, it has been shown that other problems such as impaired immunity, altered hypothalamic pituitary–adrenocortical activity, and a pro-inflammatory gene expression profile are linked to increased mortality in this patients (15).

Another issue that needs to be considered is that of increased psychological morbidity. Pshychotropic drug use is on the increase during the Covid-19 pandemic (16). It is known that isolated older people are more likely to overuse medication such as antidepressants, anxiolytics, sedatives, or hypnotics (17). Chronic overuse of hypnotics and anxiolytics can lead to confusional states, is linked to dementia (18), and is associated with falls that can lead to hip fractures, head injuries, and even death (19).

Evidence is now emerging that the Covid-19 pandemic has widespread negative effects on cognitive abilities and on the mental well-being of older people (4, 20, 21). In addition, loneliness is significantly associated with a reduction in brain volume, particularly in areas associated with memory such as the left medial temporal lobe (22). Other problems include poor sleep patterns and a decline in executive function (15), all of which increase the risk of mortality in older people.

When thinking of socially isolated people, we tend to exclude people who live in institutional settings. However, people, and particularly people with dementia, living in institutional settings (residential and nursing homes) have suffered the strongest additional negative impact both due to the ban of external visitors, but also internal social distancing measures (guidelines to keep patients in their rooms instead of letting them socialize freely in common living areas, placing patients in single occupancy rooms when possible etc. (23)). Psychiatric symptoms, leading to overuse of psychiatric medications and negative side-effects such as tardive dyskinesia and akathisia, have also increased, accelerating the deterioration of these patients and leading to complications and death (24).

It is also notable that elderly people hospitalized with Covid-19 have negative short and long -term effects that impair their functionality because of the associated fatigue, muscle weakness and sarcopenia that can exacerbate frailty, dependence and disability, making this vulnerable group even more vulnerable leading to a vicious cycle of isolation and dependence (25).

## The Cyprus Experience

Considering that cardiovascular disease is the leading cause of death in Cyprus, it is particularly important to realize that social isolation and loneliness may cause significant morbidity and mortality in these individuals. During 2018 there were 5,768 (all causes) deaths in Cyprus. Of these, 3,829 were people aged 75 years and older (26). The leading causes of death in Cyprus for the year 2017 were ischemic heart disease, stroke, Alzheimer's disease, lung cancer, diabetes, and chronic obstructive pulmonary disease (27). All of these conditions have enormous significance for older people as they are negatively affected by social isolation. Estimates of isolation-related mortality vary, with Hazzard Ratios (HR) of 1.26–3.7 (28). Even if we consider the lowest HR estimates we should expect substantial increases in the numbers of excess deaths of older Cypriot people, from causes other than Covid-19.

In addition to morbidity and mortality due to social isolation, there is an issue of extra deaths due to other effects of the lockdowns such as delayed diagnoses and lack of suitable follow-up for conditions other than Covid-19 (29). For instance, it is known that cancer risk in these situations is a real concern, particularly due to delayed diagnosis (30). We could apply the findings of this study to cancers in Cyprus. A total of 1,325 deaths occurred in Cyprus due to cancers in 2016 (31). Based on an approximate estimate of increased deaths by 10% (30), we could expect ~130 extra deaths in Cyprus per year due to delayed cancer diagnosis alone. As reduced contact with healthcare staff contributes to higher risk, efforts to address this issue are likely to lead to improved health outcomes. Therefore, there is an urgent need to implement preventative strategies for physical and mental health that may reduce death risk in older populations (21).

## Measures to Mitigate Risks

It is necessary to devise strategies for supporting older people at risk from social isolation and health deterioration. A series of interventions by the State in association with volunteers could provide such support for vulnerable older people. Initiatives that would be beneficial include the following:

- A reformed Government policy is urgently needed to allow relatives and carers of isolated older individuals to visit them (whether at home, in a care home or hospital) in order to attend to their basic psychological and physical health needs and help to prevent their deterioration. As Covid-19 PCR tests are widely available and have sensitivity of 71–98% (32), there is no reason why visitors with no Covid-19 symptoms and a negative PCR cannot meet vulnerable individuals. In cases where extra reassurance is desired, a second negative PCR test is known to confer the highest accuracy (32). In addition, spreading population strategies in favor of vaccination against Covid-19 offers glimpses of hope to reassess isolation protocols.
- Primary care services can maintain regular contact with vulnerable individuals at high risk in the community, via telephone calls from healthcare professionals in order to ensure early identification of, and intervention for, medical and other needs. The calls should be initiated by the health professional and not by the patient (33). Likewise, provision of specialist psychological support can have beneficial outcomes (34). Furthermore, volunteers, friends, and relatives can enable meaningful and supportive telephone conversations on a regular basis (35).
- Promote physical activities, preferably a mixture of resistance, strength, and balance exercises as even light intensity exercise has positive health outcomes (36). For some individuals, technology such as use of the internet, social media sites, and media broadcasts can support these exercises programs (37). Some of these programs could be presented without charge by television stations within their sphere of corporate social responsibility.
- Consider “hidden” negative aspects of issues affecting older people, such as for example, the social stigma associated with Covid-19, negative perpetuating factors promoting the pandemic, and general fear (38).
- Smart ICT solutions, such as ReMember-Me (39). These may help prevent and detect cognitive decline, promote cognitive function and social inclusion among older adults. Smart solutions offer an innovative paradigm to improve cognition, emotional well-being, activity, sleep patterns and online socialization, promoting interactions in the context of cognitive fitness and individualized suggestions for a healthy brain.
- Digital technologies can be harnessed further, and opportunities include online social and entertainment activities, networking, religious services, and board games (40), as well as cognitive training exercises and video-games (41). However, there is an issue of inequality with regards access to such technologies, and not all able, older individuals can participate (42).

## Conclusion

Social isolation due to Covid-19 restrictive measures, has been shown to have a negative impact on health outcomes, with increasing morbidity and mortality among older people. It is imperative to develop nationwide strategies to prevent adverse outcomes, other than those related to Covid-19, in socially isolated people. In our attempt to prevent Covid-19 related deaths, we should not cause additional morbidity and deaths resulting from the isolation measures we have instigated. A wide range of interventions can be implemented that can promote social engagement and interactions, improve physical functions and psychological well-being, maintain optimal health status, and prevent deterioration. Although the examples of such interventions are best suited to the older people in Cyprus, the basic principles can be applicable to older people living in any country.

## Data Availability Statement

The original contributions presented in the study are included in the article/supplementary material, further inquiries can be directed to the corresponding author/s.

## Author Contributions

MK conceptualized and wrote the manuscript. GM, HP, and MP edited, revised, and added to the manuscript. BP edited and revised the manuscript and enhanced the concept. All approved the final version of the manuscript.

## Conflict of Interest

The authors declare that the research was conducted in the absence of any commercial or financial relationships that could be construed as a potential conflict of interest.

## Publisher's Note

All claims expressed in this article are solely those of the authors and do not necessarily represent those of their affiliated organizations, or those of the publisher, the editors and the reviewers. Any product that may be evaluated in this article, or claim that may be made by its manufacturer, is not guaranteed or endorsed by the publisher.
